# Rice Germosprout Extract Protects Erythrocytes from Hemolysis and the Aorta, Brain, Heart, and Liver Tissues from Oxidative Stress* In Vitro*

**DOI:** 10.1155/2016/9587020

**Published:** 2016-06-16

**Authors:** Shahdat Hossain, Sujan Bhowmick, Marzan Sarkar, Mehedi Hassan, Jakir Hussain, Saiful Islam, Hussain Shahjalal

**Affiliations:** Laboratory of Alternative Medicine and Behavioral Neurosciences, Department of Biochemistry and Molecular Biology, Jahangirnagar University, Savar, Dhaka 1342, Bangladesh

## Abstract

Identifying dietary alternatives for artificial antioxidants capable of boosting antihemolytic and antioxidative defense has been an important endeavor in improving human health. In the present study, we studied antihemolytic and antioxidative effects of germosprout (i.e., the germ part along with sprouted stems plus roots) extract prepared from the pregerminated rice. The extract contained considerable amounts of antioxidant *β*-carotene (414 ± 12 ng/g of extract) and phytochemicals such as total polyphenols (12.0 ± 1.1 mg gallic acid equivalent/g of extract) and flavonoids (11.0 ± 1.4 mg catechin equivalent/g of extract). The antioxidant potential of the extract was assessed by its DPPH- (2,2-diphenyl-1-picrylhydrazyl-) free radical scavenging activity where we observed that germosprout extract had considerable antioxidative potentials. To evaluate antihemolytic effect of the extract, freshly prepared erythrocytes were incubated with either peroxynitrite or Fenton's reagent in the absence or presence of the extract. We observed that erythrocytes pretreated with the extract exhibited reduced degree of* in vitro* hemolysis. To support the proposition that germosprout extract could act as a good antioxidative agent, we also induced* in vitro* oxidative stress in erythrocyte membranes and in the aorta, brain, heart, and liver tissue homogenates in the presence of the extract. As expected, germosprout extract decreased oxidative stress almost to the same extent as that of vitamin E, as measured by lipid peroxide levels, in all the mentioned tissues. We conclude that rice germosprout extract could be a good natural source of antioxidants to reduce oxidative stress-induced hemolysis and damage of blood vessels and other tissues.

## 1. Introduction

Oxidative stress is essentially an imbalance between the production of free radicals and the ability of the body to counteract or detoxify their harmful effects through neutralization by its own antioxidants [1, 2]. Oxidative stress has been reported as the contributing factor in various diseases such as inflammatory diseases, ischemic heart diseases, hypertension, hypercholesterolemia, stroke, various liver diseases, hemochromatosis, neurodegenerative disorders, and smoking-related diseases [3–8] and in the impairment of erythrocyte functions including hemolysis [9]. An excess of oxidative stress can lead to the oxidation of lipids and proteins, which is associated with changes in their structures and functions. The recent growth in the knowledge of free radicals and their detrimental effects on human health is leading a medical revolution, promising a new era of disease management [10–13]. Ironically, oxygen, an element indispensable for life, under certain situations has deleterious effects on the human body [12, 13]. Most of the potentially harmful effects of oxygen are due to the formation of a number of chemical compounds, known as reactive oxygen species (ROS) or reactive nitrogen species (RNS), which have a tendency to donate electron to other cellular components. Recently, study of free radicals and antioxidants has provided important understanding of disease mechanisms, thus paving the way for their prevention and treatments [12, 13]. However, synthetic antioxidants have been reported to be harmful to human health [4]. Therefore, in addition to endogenous antioxidant defense systems, consumption of dietary and plant-derived antioxidants could be a suitable alternative. A large body of evidences from both epidemiologic and biochemical studies clearly demonstrates that dietary intake of antioxidants plays a protective role in human health, decreasing the incidence of several diseases in different populations [14–16]. Thus, the search for effective and nontoxic natural foods or compounds with antioxidative activity has been intensified in recent years.

Many different health benefits have been associated with sprouted grains [17]. Rice is an important staple starchy food consumed by more than half of the world population [18]. The demand for pregerminated types of rice is increasing day by day due to their high content of vitamins, minerals, and dietary fibers; and it is a healthy preventative measure for hypertension, diabetes, hypercholesterolemia, and a host of other cardiovascular disease-related complications. Hsu et al. reported that brown pregerminated rice had decreased fasting blood glucose, as well as blood levels of total cholesterol and fructosamine in type 2 diabetic humans [19]. Pregerminated rice is also reported to decrease plasminogen activator inhibitor-I in streptozotocin-induced diabetic model rats [20]. Rats fed with sprouted rice presented increased plasma level of HDL-cholesterol [21]. Furthermore, the sprouted brown rice-fed lactating mothers exhibited decreased scores of depression, anger-hostility, and fatigue, indicating that pregerminated rice may have beneficial effects on psychosomatic health [22]. Sprouted wheat was also reported to protect against fatty liver [23]. Besides, rats fed with germinated wheat had lower systolic blood pressure and exhibited significantly reduced oxidative damage in aortic endothelial cells [24]. Converging evidences thus indicate that the benefits of sprouted rice intake on human health should be ascribed not only to the hypoglycemic and hypolipidemic effects but also to the antioxidant properties.

In this study, we allowed the mature rice seeds to be sprouted under suitable condition in our laboratory, isolated the germosprout, that is, the germ plus sprouted roots and stem parts of the rice, and then dried, powdered, and prepared hot water extract to examine whether it could exhibit any protective effect against free radical-induced hemolysis of the erythrocytes. Additionally, we evaluated the antioxidative defense of germosprout extract against free radical-induced oxidative stress in several tissues of the rats, in particular, the aorta, brain, heart, and liver tissues* in vitro*. Our results provide evidence that germosprout extract of the pregerminated rice possesses considerable amounts of antioxidant phytochemicals (polyphenols, flavonoids) and an antioxidant vitamin (*β*-carotene), which might prevent free radical-induced hemolysis of the erythrocytes, as well as oxidative stress-associated damage of the tissues* in vitro*.

## 2. Materials and Methods

### 2.1. Chemicals

1,1,3,3-Tetraethoxypropane (TEP), bovine serum albumin (BSA), peroxynitrite, hydrogen peroxide (H_2_O_2_), and FeSO_4_ were purchased from Sigma Aldrich (St. Louis, USA). Absolute ethanol was purchased from Hong Yang Chemical Corporation (China). Folin reagent and sodium dodecyl sulphate were purchased from Merck (Darmstadt, Germany).

### 2.2. Preparation of Germosprout Powder from Pregerminated Rice

**Figure 1:**
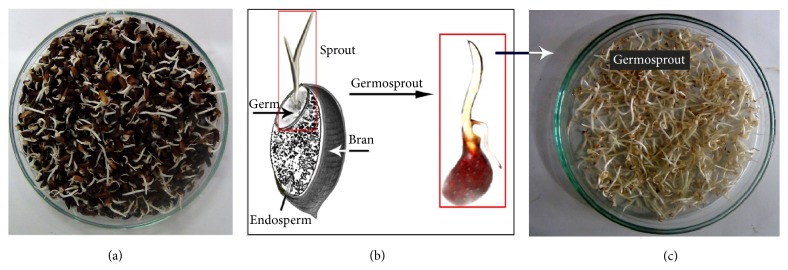
(a) Sprouted rice taken in the Petri dish. (b) depicts different parts of sprouted rice and (c) germosprouts (germs plus sprouted stems and roots) collected for use in the experiments.

Mature rice (*Oryza sativa* L.) seeds (5 kg) of* Kalijira* variety were purchased from a local farmer at Comilla district, Bangladesh, who has been traditionally storing and cultivating rice for years. Germination was carried out according to the method of Roy et al. [25]; however, it was modified and adapted to our laboratory. Rice seeds were soaked in tap water at room temperature for 24 h and water was changed approximately after every 8 h. The soaked rice seeds were then distributed onto a handmade open tray. The seeds were covered by a thick jute cloth and then laboratory germination was carried out for 72 h at 28–30°C. When both the plumule and radicle extended to more than 1.0 cm, the germosprouts (i.e., the germ part along with sprouted stems plus roots) were separated manually, as shown in Figure 1. The germosprouts were dried at 37°C for 24 h before being ground into fine powder.

### 2.3. Estimation of Total Protein in Germosprout Powder

The fine ground powder was soaked with 0.1 N NaOH in glass screw capped test tubes for 24 h with brief sonication in an ultrasound bath sonicator filled with ice water (at maximum output). Then, the test tubes were vortexed and heated at 80°C in block heater for 2 min. After centrifugation at 2000 ×g, total protein was measured from the supernatant by Lowry method [26] and was calculated as mg of protein per gram of germosprout powder.

### 2.4. Estimation of Total Lipid in Germosprout Powder

Total lipid content of germosprout powder was estimated gravimetrically. Total lipid was extracted according to the method of Folch et al. [27]. The fine ground powder was soaked in glass screw capped test tubes with a solution consisting of chloroform : methanol (2 : 1) for 24 h with brief sonication in an ultrasound bath sonicator filled with ice water (at maximum output). Then, the test tubes were vortexed and incubated at 4°C for 24 h. After centrifugation at 2000 ×g, the chloroform layer was collected. The procedure was repeated two more times. All chloroform layers were combined together and evaporated to dryness. The amount of total lipid was calculated from the pre- and postweights of the test tubes and expressed as mg of lipid per gram of germosprout powder.

### 2.5. Estimation of *β*-Carotene Content

*β*-Carotene content was determined as described by Nagata and Yamashita [28] with slight modification. The dried germosprout powder (100 mg) was vigorously shaken with 10 mL solution consisting of acetone : hexane (4 : 6) for 10 min and then filtered through Whatman number 4 filter paper. The absorbance of the filtrate was measured at 453, 505, 645, and 663 nm spectrophotometrically. *β*-Carotene content was calculated according to the following equation: *β*-carotene (mg/100 mL) = 0.216 × *A*_663_ − 1.22 × *A*_645_ − 0.304 × *A*_505_ + 0.452 × *A*_453_. Finally, the concentration of *β*-carotene was expressed as ng of *β*-carotene/g of germosprout extract.

### 2.6. Estimation of Total Polyphenol and Flavonoid Contents

Total polyphenol content of germosprout extract was determined by Folin and Ciocalteu's method against gallic acid standard as previously described [29] and the concentration in the extract was expressed as gallic acid equivalents (*μ*g of gallic acid/mg of extract). Total flavonoid content of germosprout extract was measured by aluminum chloride colorimetric assay against quercetin standard [29] and its concentration in the extract was expressed as quercetin equivalents (*μ*g of quercetin/mg of extract).

### 2.7. Preparation of Germosprout Extract for Antioxidant Activity

The germosprout fine powder was extracted with boiling water for 30 minutes and allowed to steep with continuous swirling. Extracts were filtered through Whatman number 1 filter paper, centrifuged at 2000 ×g for 30 min, aliquoted, and stored at −20°C for analyses.

### 2.8. DPPH-Free Radical Scavenging Activity

DPPH radical scavenging activity was measured by determining the decrease in absorbance of methanolic DPPH solution at 517 nm in the presence of germosprout extract, as previously described [9]. In brief, the hot water extract was diluted with methanol. 100 *μ*L of the diluted extract with different concentrations was added to 100 *μ*L of 0.4 mM methanolic solution of DPPH. The reaction mixtures were vortexed and allowed to stand for 30 min at room temperature in the dark before the absorbance at 517 nm was measured using methanol as blank. Free radical scavenging activity was expressed as IC_50_, that is, concentration of the extract required to decrease the absorbance of DPPH (0.2 mM, final concentration) by fifty percent. The unit for the concentration of the extract was adjusted to *μ*g of the germosprout powder/mL of hot water extract.

### 2.9. Animals

Five-week-old male* Wistar* rats purchased from icddr,b (Dhaka, Bangladesh) were housed in an air-conditioned animal room with a 12 : 12 h dark : light cycle under controlled temperature (23 ± 2°C) and humidity (50 ± 10%). The rats were provided with a normal pellet diet with water* ab libitum*. All animal experiments were performed in accordance with the procedures outlined in the Guidelines for Animal Experimentation of Jahangirnagar University compiled from the Guidelines for Animal Experimentation of the Bangladesh Association for Laboratory Animal Science.

## 3. Preparation of Erythrocytes and Erythrocyte Ghost Membranes

After deep anesthesia with pentobarbital blood from individual rat was collected from the inferior vena cava with a heparinized syringe. Then, erythrocytes were isolated from the blood as described by Hashimoto et al. [30]. The resultant purified erythrocytes were subjected to hemolysis or used for preparation of erythrocyte ghost membranes as described previously [9].

### 3.1. *In Vitro* Hemolysis Assay

The extent of erythrocyte hemolysis was determined as described previously [9, 31]. In brief, erythrocyte suspensions at 2% hematocrit were incubated with peroxynitrite (at 100 *μ*M final concentration) or with freshly prepared Fenton's reagent [H_2_O_2_ (45 mM) + FeSO_4_ (2 mM)] at 37°C for 1 h. At the end of incubation, erythrocytes were pelleted down by centrifuging the samples at 300 ×g for 10 min. Then, the supernatant was aspirated and the extent of hemolysis was quantified by determining the amounts of released hemoglobin (Hb) in the supernatant at 540 nm against hemoglobin standard.

### 3.2. Antihemolytic Effect of Germosprout Extract* In Vitro*

Erythrocyte suspensions at 2% hematocrit were incubated with peroxynitrite (at 100 *μ*M final concentration) or with freshly prepared Fenton's reagent in the absence or presence of 100 *μ*L of germosprout extract (10 mg/mL) at 37°C for 1 h. At the end of incubation, the extent of hemolysis was determined against hemoglobin standard, as described above.

### 3.3. Preparation of Tissue Homogenates

After drawing blood, rats were initially perfused with ice-cold saline to remove blood from the brain. Then, the aorta, brain, heart, and liver were separated from rats, reperfused with saline, and homogenized in phosphate buffer (100 mM, pH 7.4) containing 1% phenylmethylsulfonyl fluoride (PMSF). The homogenate was centrifuged at 1000 ×g to remove unbroken tissues and debris and the resultant homogenates were assigned as aorta, brain, heart, or liver tissue homogenates, respectively, which were stored at −20°C until analysis.

### 3.4. Antioxidative Stress Activity of Germosprout Extract* In Vitro*

Antioxidative stress activity of germosprout extract was evaluated by determining the levels of lipid peroxide (LPO) in erythrocyte membranes and in the aorta, brain, heart, and liver tissues* in vitro*. Oxidative stress was directly induced in erythrocyte membranes and in the above tissue homogenates by Fenton's reagent, as described previously [9]. For the determination of antioxidative stress activity, erythrocyte membranes and the above tissue homogenates were divided into (1) tissue homogenate (0.1 mL) alone (control); (2) tissue homogenate plus Fenton's reagent (OS); and (3) tissue homogenate plus Fenton's reagent (OS) plus 100 *μ*L of germosprout extract (10 mg/mL) (OS + GSE), all of which were incubated at 37°C for 2 h. Then, the levels of LPO were measured to examine whether germosprout extract exhibits any effects on production of LPO, that is, the effects against the oxidative stress* in vitro*. In addition, vitamin E (100 *μ*L, 12.5 mM (final concentration)) was added separately with the tissue homogenates along with Fenton's reagent (OS + Vit E) to compare the antioxidative activities of germosprout extract.

### 3.5. Lipid Peroxide (LPO) Assay

The basal LPO levels in erythrocyte membranes and the aorta, brain, heart, and liver tissues were determined by estimating the thiobarbituric acid reactive substance (TBARS), as described previously by Hashimoto et al. [32]. In brief, erythrocyte membranes and the aorta, brain, heart, and liver tissue homogenates (0.1 mL) from rats were added to 0.1 mL of 8.1% (w/v) sodium dodecyl sulphate, 2 mL of 0.4% thiobarbituric acid in 20% acetic acid (pH 3.5), and 0.1 mL distilled water. Each tube was tightly capped and heated at 95°C for 1 h. After cooling the tubes with tap water, 2 mL of n-butanol-pyridine (15 : 1, v/v) was added and shaken vigorously for about 10 minutes. The tubes were then centrifuged at 1200 ×g for 10 minutes at room temperature (digital centrifuge; DSC-1512SD). The absorbance of the upper organic layer was measured at 532 nm. The TBARS levels were measured in nmoles of malondialdehyde (MDA)/mg of protein. MDA levels were calculated relative to a standard preparation of 1,1,3,3-tetraethoxypropane (TEP). Protein concentration was measured by Lowry method [26].

### 3.6. Statistics

All results were expressed as mean ± standard error of the mean (SEM). The significance of difference in means among different groups for antihemolytic or antioxidative stress activity was determined by one-way ANOVA, followed by Fisher's PLSD test for* post hoc* comparisons using GraphPad Prism® software version 5.0 (GraphPad Software Inc., San Diego, CA, USA). *P* < 0.05 was considered statistically significant.

## 4. Results

### 4.1. Total Protein, Total Lipid, *β*-Carotene, and Antioxidant Phytoconstituents of Germosprouts

Total protein content of the germosprouts was 97.2 ± 4.2 mg/g of powder, while total lipid content was 24.0 ± 2.8 mg/g of powder. The *β*-carotene content was 414 ± 12 ng/g of extract. In addition, germosprout extract also had considerable amounts of antioxidant phytochemicals, such as total polyphenols (12.0 ± 1.1 mg gallic acid equivalent/g of extract) and total flavonoids (11.0 ± 1.4 mg catechin equivalent/g of extract). These findings suggest that the germosprouts of pregerminated rice could be used as a good source of antioxidants.

### 4.2. DPPH-Free Radical Scavenging Activity of Germosprout Extract

**Figure 2:**
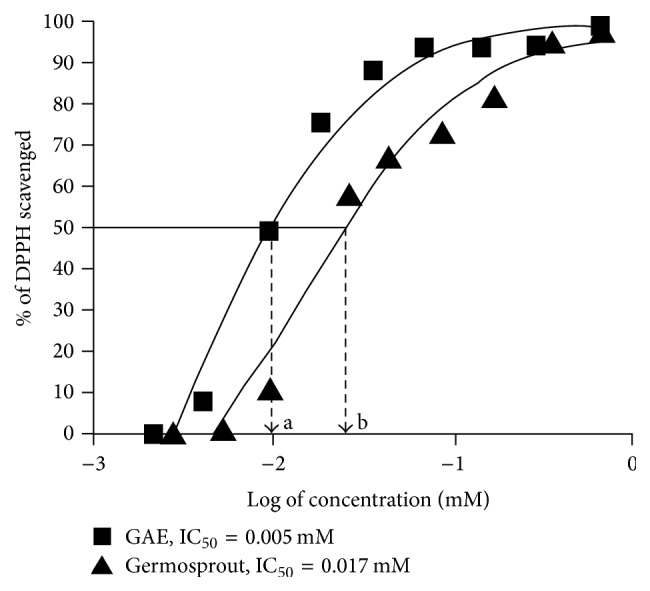
Germosprout extract scavenged DPPH-free radical. The figure illustrates the ability of germosprout extract and gallic acid to scavenge DPPH-free radicals. Data are the representative of triplicate determinations. Data were subjected to nonlinear sigmoidal dose-response curve, *Y* = bottom + (top − bottom)/(1 + 10^(log⁡IC_50_ − *X*)^), where *X* is the logarithm of concentration. *Y* = the scavenging response. *Y* starts at the bottom and goes with sigmoid shape. Bottom = *Y*_minimum_ (0), top = *Y*_minimum_ (100%), and log⁡IC_50_ = the value of *X* at *Y*-mid. In other words, IC_50_ is the concentration of antioxidants (here, gallic acid and germosprout extract containing antioxidants such as polyphenols) required to reach half-maximal scavenging of DPPH-free radical. GAE: gallic acid equivalent.

Due to the presence of antioxidant components in germosprout extract, we checked whether this extract has DPPH-free radical scavenging ability or not (Figure 2). We observed that germosprout extract was able to reduce the purple color of DPPH and abolished the absorption peak at 517 nm, indicating DPPH-free radical scavenging ability of the extract. Results also showed that DPPH-free radical scavenging activity was increased with the increase in the concentration of germosprout extract (Figure 2). However, the IC_50_ for germosprout extract was 0.0017 mM (GAE) (equivalent to ~0.3 mg/mL of extract) and that of control (gallic acid) was 0.005 mM, indicating that the IC_50_ was one-third to that of the standard gallic acid at the concentration of the extract used. These results indicate that germosprout extract, which contains antioxidants, possesses considerable antioxidative potential.

### 4.3. Protective Effects of Germosprout Extract against Erythrocyte Hemolysis* In Vitro*

**Figure 3:**
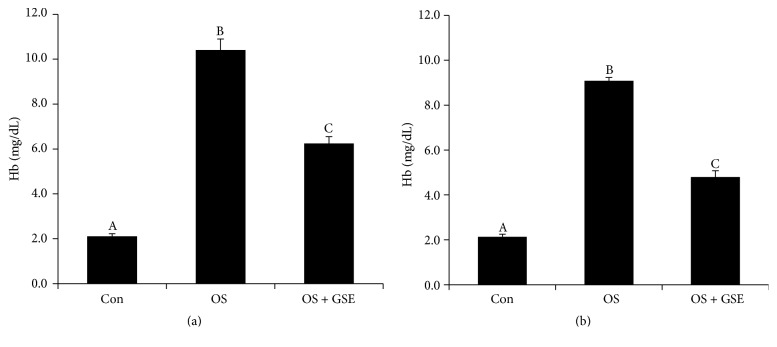
Effects of germosprout extract on (a) peroxynitrite- and (b) Fenton's reagents-induced hemolysis of erythrocytes* in vitro*. Results are expressed as mean ± SEM (*n* = 5), each with duplicate determinations. Bars with different letters are significantly different at *P* < 0.05. Data were analyzed with one-way ANOVA followed by Fisher's PLSD for* post hoc* comparison. Hb: hemoglobin, OS: oxidative stress, and GSE: germosprout extract.

Mammalian erythrocytes are a unique and interesting cellular model for research on oxidative stress, induced by either reactive nitrogen species (RNS) or reactive oxygen species (ROS), as well as for studies on the molecular mechanisms underlying the protective effects of the antioxidants. Therefore, we investigated whether germosprout extract possesses any protective effects against oxidative stress-induced hemolysis* in vitro*. For this purpose, erythrocytes were incubated with germosprout extract and oxidative stress that leads to hemolysis was induced simultaneously by addition of either peroxynitrite or Fenton's reagent. Results showed that incubation of erythrocytes in the presence of peroxynitrite or Fenton's reagent resulted in an extensive hemolysis (Figure 3), suggesting that both peroxynitrite and Fenton's reagent are almost similarly effective for inducing erythrocyte hemolysis* in vitro*. Most importantly, erythrocytes pretreated with the extract had reduced degree of peroxynitrite- or Fenton's reagent-induced hemolysis, confirming that germosprout extract effectively protects erythrocytes from oxidative stress-induced hemolysis* in vitro*. We also observed that germosprout extract itself did not cause lysis of any erythrocytes* in vitro* (data not shown).

### 4.4. Germosprout Extract Reduces Oxidative Stress in Erythrocyte Membranes and in the Aorta, Brain, Heart, and Liver Tissues* In Vitro*

**Figure 4:**
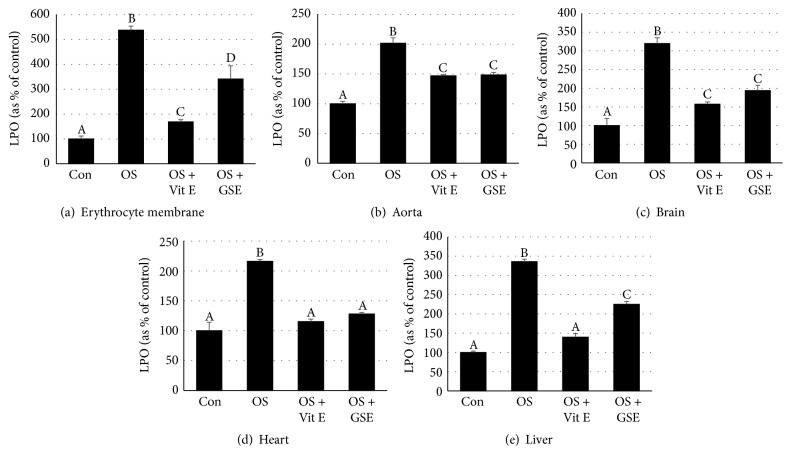
Effects of germosprout extract on Fenton's reagent-induced oxidative stress in erythrocyte membranes (a) and in the aorta (b), brain (c), heart (d), and liver (e) tissues. For erythrocyte membranes or a tissue, the bars with different letters are significantly different at *P* < 0.05. Data were subjected to one-way ANOVA followed by Fisher's PLSD* post hoc* test for multiple comparisons. LPO: lipid peroxide, Con: control, OS: oxidative stress, OS + Vit E: OS + vitamin E (*α*-tocopherol), and OS + GSE: OS + germosprout extract.

We next induced oxidative stress in erythrocyte membranes as well as in several tissue homogenates particularly in the aorta, brain, heart, and liver tissue homogenates* in vitro* with the use of Fenton's reagent and examined whether oxidative stress in these tissues could be inhibited by germosprout extract. Lipid peroxide (LPO) levels were measured in these tissue samples as indicator of oxidative stress in the absence or presence of germosprout extract. Moreover, we compared antioxidative defense of germosprout extract with that of an antioxidant vitamin, *α*-tocopherol (i.e., vitamin E). As expected, Fenton's reagent-induced oxidative stress significantly increased LPO levels in erythrocyte membranes, as well as in the aorta, brain, heart, and liver tissues (Figure 4). In contrast, LPO levels significantly reduced in erythrocyte membranes, as well as in the above tissues when these were pretreated with either germosprout extract or vitamin E. However, in the aorta, brain, and heart tissues, germosprout extract exhibited similar antioxidative defense (as indicated by the LPO levels) to that of vitamin E, while, in erythrocyte membranes and liver tissues, the effects were lower than that of vitamin E. All these results collectively suggest that germosprout extract had significant effect in inhibiting oxidative stress not only in erythrocyte membranes but also in other body tissues such as the aorta, brain, heart, and liver tissues.

## 5. Discussion

The rice grains are seeds of paddy plants. Parts of the whole grain—the germ, the endosperm, and the bran—are crucial to creating a new plant. The germ is the plant embryo, which when it grows will feed on the starchy endosperm. The germ imparts many effects to the germinated seeds, which when consumed also confers many beneficial effects to the consumers compared to that of nongerminated rice. The purpose of this study was to investigate whether the sprouted germs (germosprouts) of pregerminated rice seeds have overwhelming effects on free radical-induced hemolysis of erythrocytes and oxidative stress in the aorta, brain, heart, and liver tissues* in vitro*. We selected a rice variety, known as* Kalijira* (miniature basmati), which is nonglutinous rice with milky smell. The germosprout extract of this rice contained considerable amounts of antioxidant phytochemicals, such as total polyphenols (12.0 ± 1.1 mg gallic acid equivalent/g of extract) and total flavonoids (11.0 ± 1.4 mg catechin equivalent/g of extract). In addition, the extract also contained significant amount of an antioxidant vitamin, *β*-carotene (414 ± 12 ng/g of extract). Hence, the germosprouts of pregerminated rice could be used as a good source of antioxidants. Our results are consistent with the previous reports where sprouting wheat displayed higher levels of antioxidant phenolic compounds than unsprouted wheat [33, 34] and hence antioxidative potentials. Due to the presence of antioxidant components, germosprout extract exhibited DPPH-free radical scavenging effect. Partial nutritional composition analysis suggests that the germosprouts contained considerable amount of protein and lipids.

In the present study, the erythrocytes treated with peroxynitrite or hydroxyl radicals (of Fenton's reagent) displayed approximately 4- to 5-fold increase in hemolysis* in vitro*. The results are consistent with our previous reports that free radicals readily cause erythrocyte hemolysis [9]. There are also* in vivo* findings which have reported that oxidative stress aggravates hemolysis [35], as seen during hemolytic anemia, beta-hemoglobinopathies, glucose-6-phosphate dehydrogenase deficiency, and dyserythropoietic anemia. Although oxidative stress is not the primary etiology of these diseases, oxidative damage to their erythroid cells, however, plays a critical role in hemolysis. Erythrocyte membrane phospholipids constitute a major target for the cytotoxic effect of RNS or ROS in that their polyunsaturated fatty acids are particularly prone to peroxidation, leading to the ultimate formation of malonaldehyde. We observed a dramatic increase in malonaldehyde concentration in erythrocyte membranes when treated with ROS (hydroxyl radicals of Fenton's reagent), indicating that severe peroxidative damage in erythrocyte membranes occurs during hemolysis. The hemolysis of erythrocytes induced by peroxynitrite or hydroxyl radicals significantly decreased when they were cotreated with germosprouts extract. This finding is consistent with the presence of antioxidants like polyphenols, flavonoids, and *α*-tocopherol in the extract; otherwise, the degree of hemolysis would have not been decreased.

The results of antihemolytic effect of germosprout extract led us to induce oxidative stress* in vitro* in several tissues, particularly the aorta, brain, heart, and liver tissues with the use of hydroxyl radical (a major product of Fenton's reagent) and investigate antioxidative defense of this extract. As expected, oxidative stress significantly reduced in these tissues in the presence of germosprout extract, further suggesting that antioxidants present in germosprout extract might be candidate compounds to confer antioxidative defense on these tissues. Our results corroborate well the previous reports that germinated rice and buckwheat extracts improve antioxidative status and protect liver and aortic endothelial cells [23, 24, 36]. Mohd Esa et al. also demonstrated a protective effect against oxidative damage in diabetic rats fed on rice bran extract [37]. In order to verify whether the effect of germosprout extract, which ultimately resulted in protection against cellular oxidative cytotoxicity, was mediated by antioxidant compounds present in the extract, we also induced oxidative stress in erythrocyte membranes and the aorta, brain, heart, and liver tissues in the presence or absence of an antioxidant vitamin, *α*-tocopherol (i.e., vitamin E). We found that vitamin E significantly inhibited oxidative stress in erythrocyte membranes, as well as in the tissues, suggesting that the protection against free radical-induced hemolysis of erythrocytes and oxidative stress in the aorta, brain, heart, and liver tissues might have been, at least partially, imparted by the antioxidants present in germosprout extract. We also observed that LPO concentrations in erythrocyte membranes and in the aorta, brain, heart, and liver tissues incubated only with germosprout extract were similar to those of control samples (data not shown), suggesting that germosprout extract itself did not have any oxidative potential.

The consequence of oxidative stress in erythrocytes can contribute to oxidative stress in artery. Conversely, free radicals such as peroxynitrite or hydroxyl radical produced by the blood vessels can easily propagate to the circulatory cells such as erythrocytes and contribute to the etiology of many chronic health problems such as hemolytic anemia and cardiovascular and inflammatory diseases [35, 38–40]. Antioxidants present in the germosprout may reduce free radical-induced hemolysis of erythrocytes, as well as oxidative stress in the blood vessels, brain, heart, and hepatic tissues by preventing formation of free radicals and/or scavenging them. Oxidative stress that leads to oxidative modifications of proteins, nucleic acids, and lipids plays an important role in brain aging, neurodegenerative diseases, and other related adverse conditions, such as ischemia [4, 7, 41–43]. The decreased level of LPO observed in germosprout extract-treated brain tissue thus indicates beneficial effects of the extract against oxidative stress-associated diseases in the brain. Several* in vitro* and* in vivo* observations suggest that oxidative stress and associated damage could represent a common link between different forms of chronic liver injury and hepatic fibrosis. For example, oxidative stress contributing to lipid peroxidation is one of the critical factors involved in the genesis and the progression of nonalcoholic steatohepatitis and liver cancer [44, 45]. Besides, oxidative stress is increasingly being recognized as a fundamental factor in the pathologic changes observed in various vascular diseases including hypertension and hypercholesterolemia [3]. Thus, inhibition of oxidative stress (indicated by reduced levels of LPO) in the aorta, heart, and liver tissues by germosprout extract suggests that it has considerable influence on various oxidative stress-instigated pathologic changes in the liver and the cardiovascular system.

## 6. Conclusion 

Oxidized macromolecules in the body result in decreased function and shortened life span. Avoiding the formation of free radicals and reducing oxidative stress, thereby strengthening the body's antioxidant defenses, can reduce the risk of oxidation-associated diseases. The primary prevention of chronic diseases through dietary modification may be just as effective as the secondary treatments that are commonly employed and less costly. In this sense, searching dietary alternatives for artificial antioxidant therapy are necessary that may yield dividends in the near future in improving health. In this study, as a part of such objectives, we evaluated germosprout extract prepared from pregerminated rice for its antihemolytic and antioxidative potentials; and our results provide evidence that germosprout extract is very beneficial, at least, from the perspective of antioxidant potential that could prevent oxidative hemolysis of erythrocytes, as well as oxidative stress-associated damage in the aorta, brain, heart, and liver tissues.
